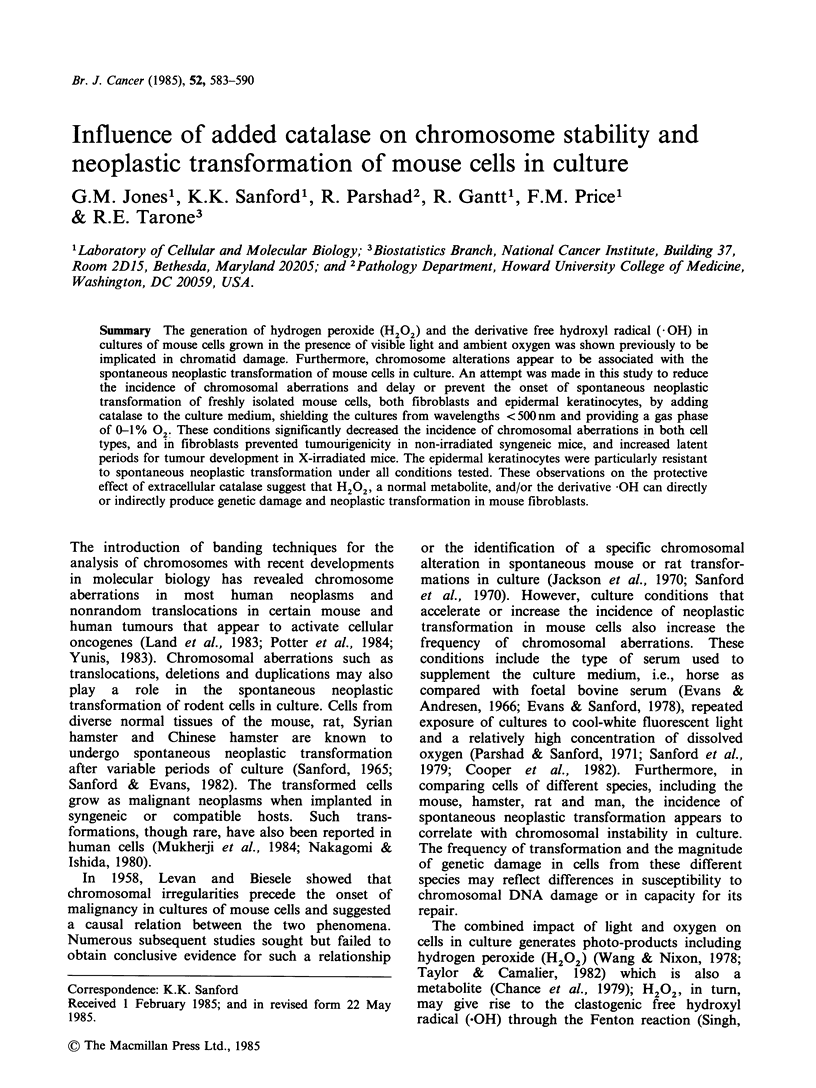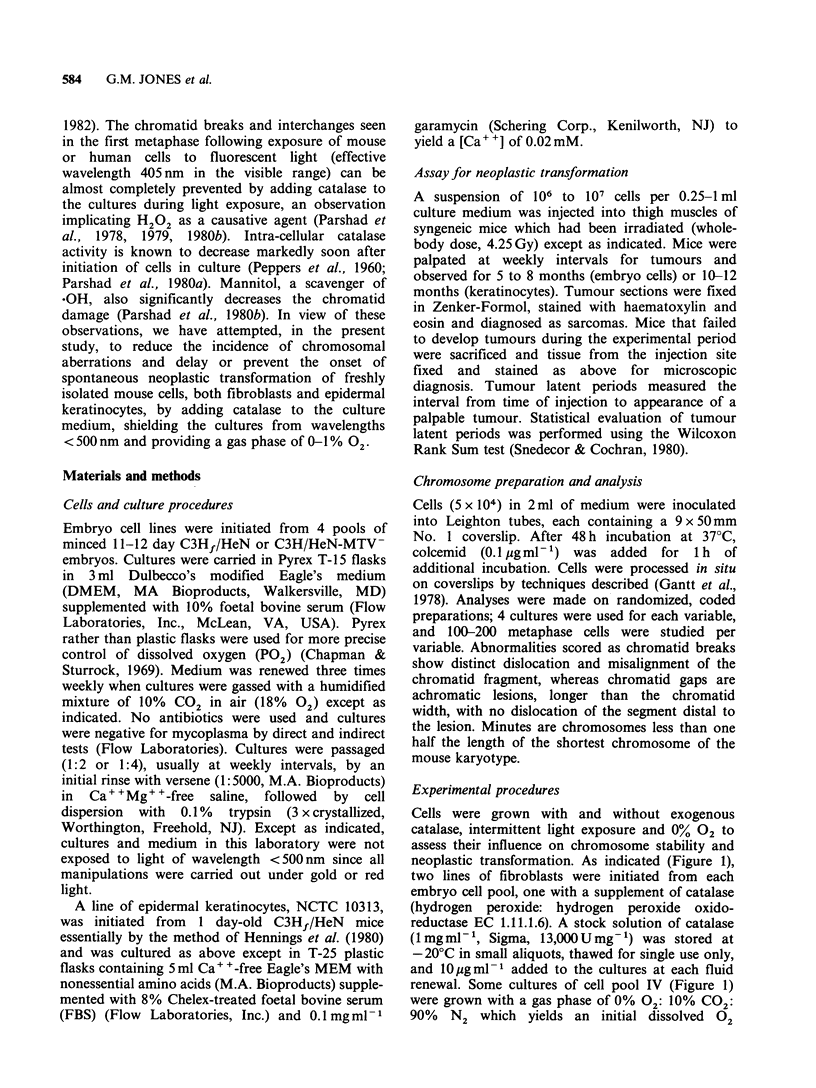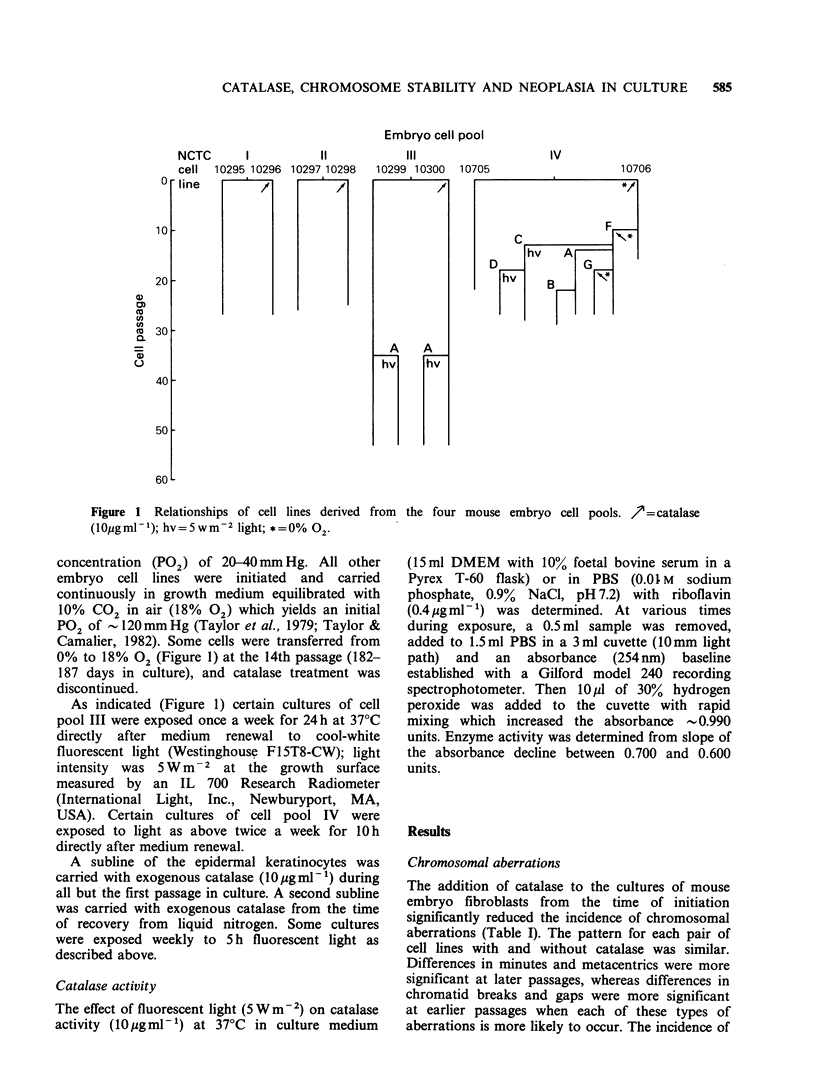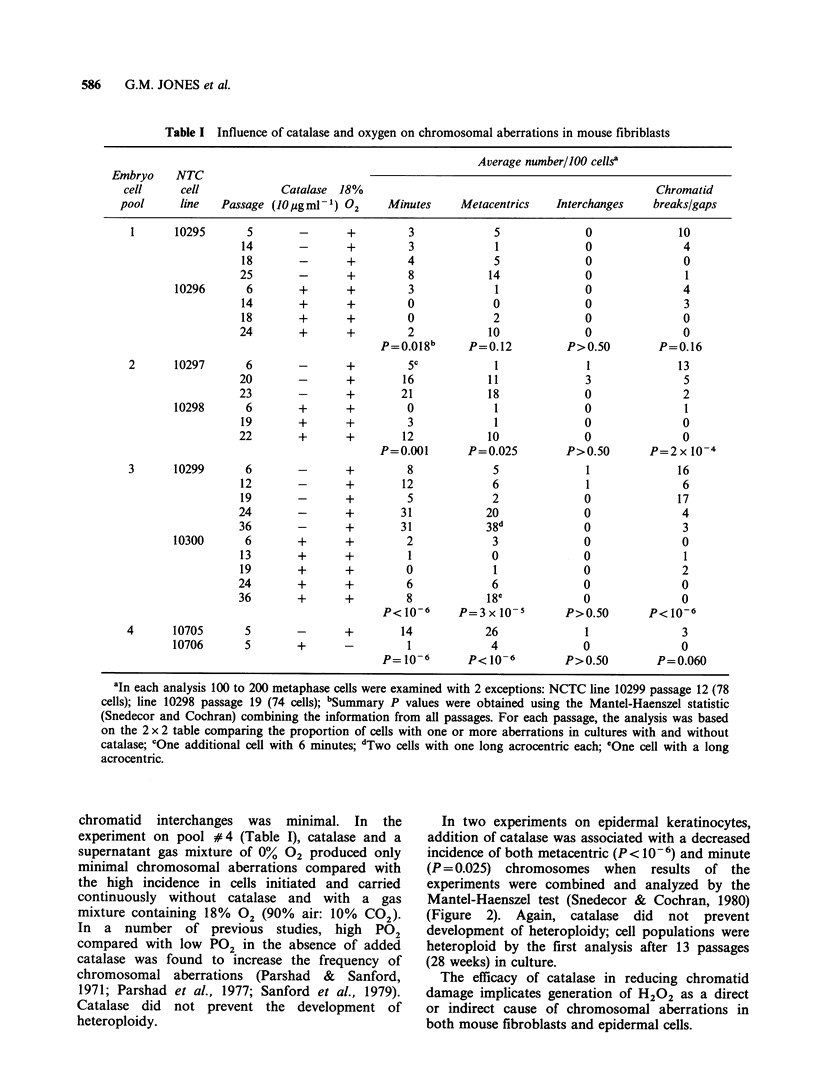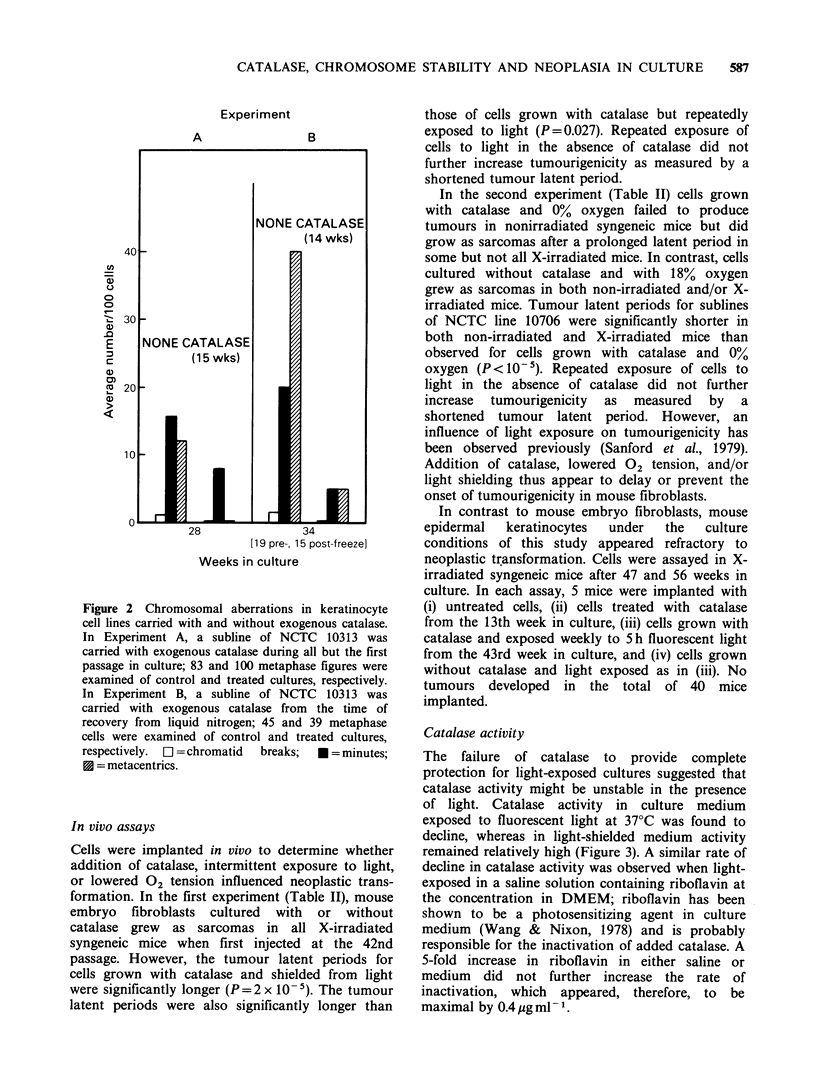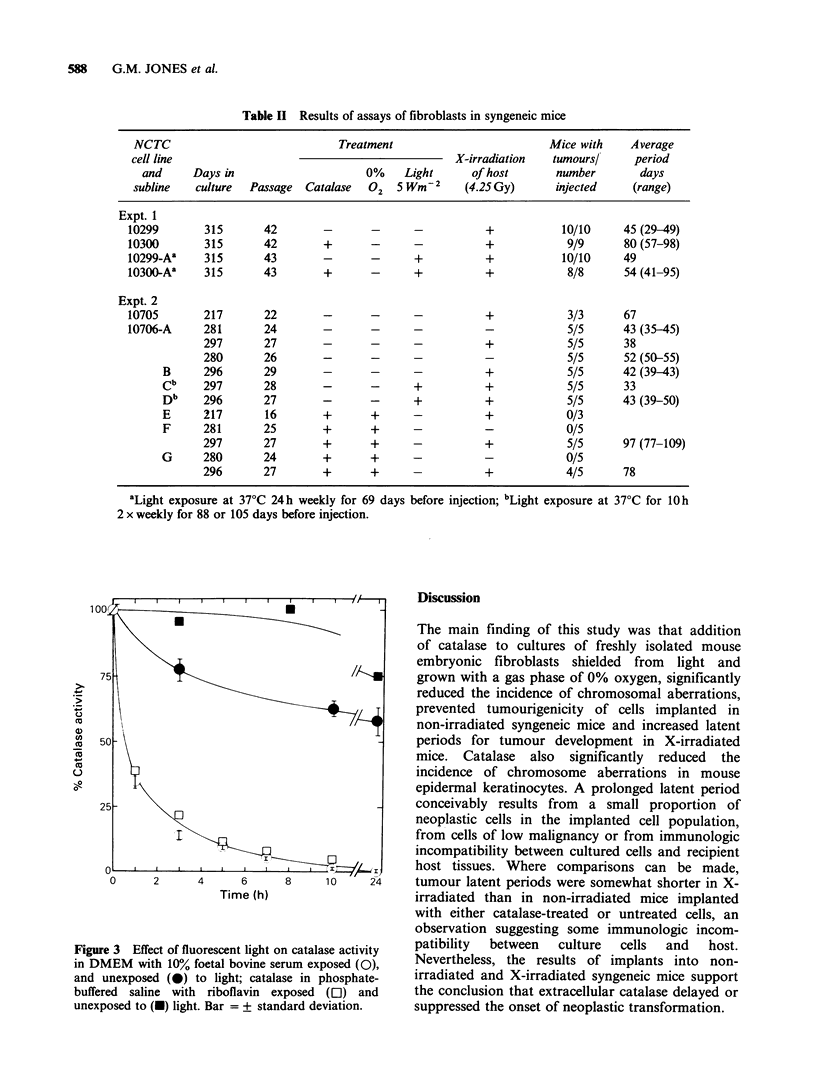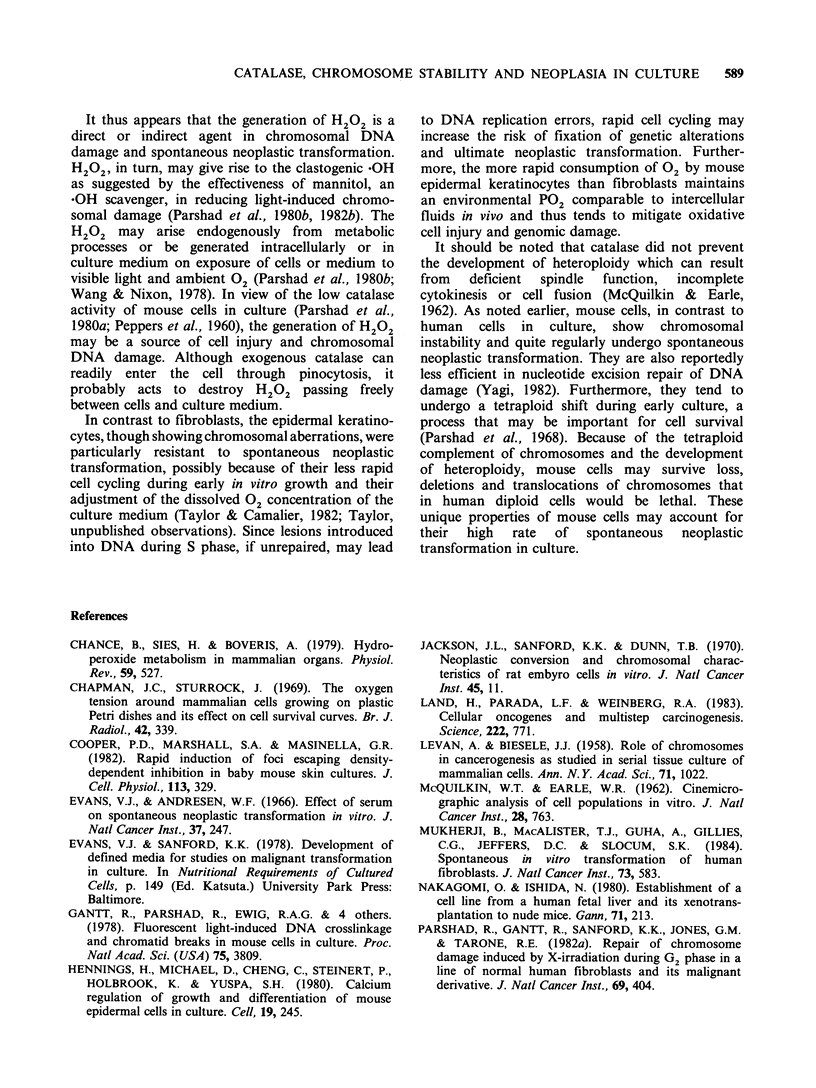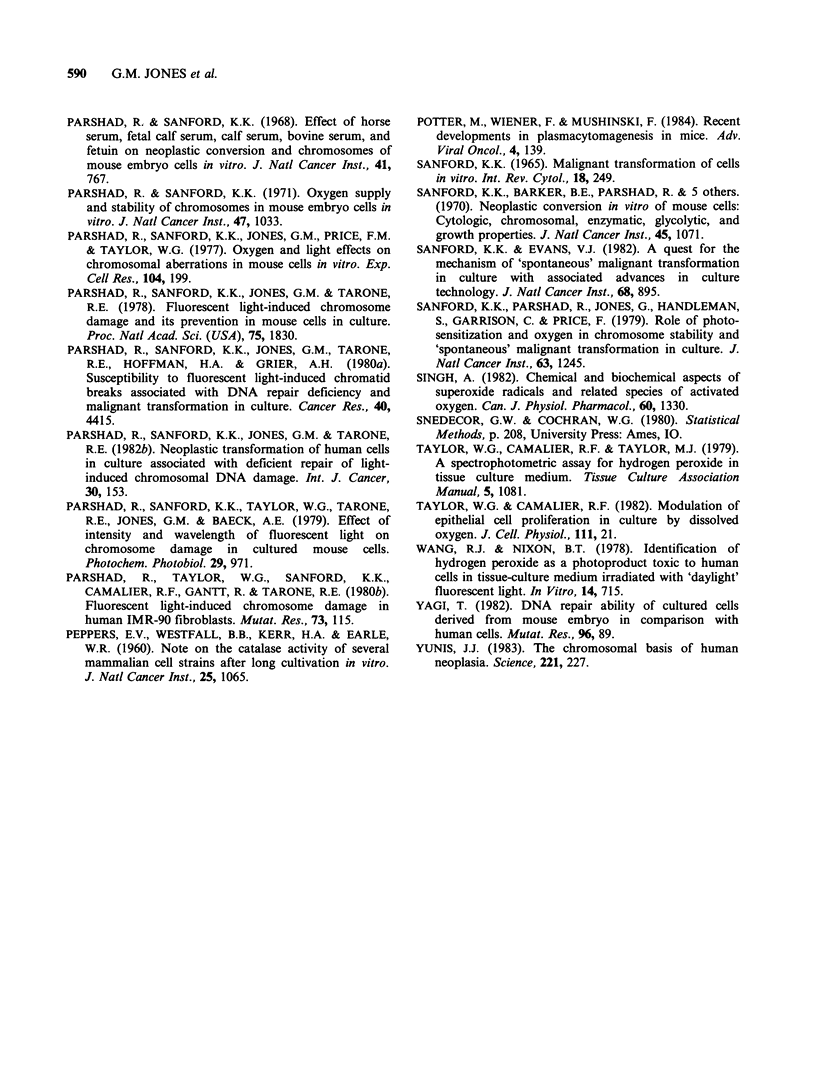# Influence of added catalase on chromosome stability and neoplastic transformation of mouse cells in culture.

**DOI:** 10.1038/bjc.1985.230

**Published:** 1985-10

**Authors:** G. M. Jones, K. K. Sanford, R. Parshad, R. Gantt, F. M. Price, R. E. Tarone

## Abstract

The generation of hydrogen peroxide (H2O2) and the derivative free hydroxyl radical (. OH) in cultures of mouse cells grown in the presence of visible light and ambient oxygen was shown previously to be implicated in chromatid damage. Furthermore, chromosome alterations appear to be associated with the spontaneous neoplastic transformation of mouse cells in culture. An attempt was made in this study to reduce the incidence of chromosomal aberrations and delay or prevent the onset of spontaneous neoplastic transformation of freshly isolated mouse cells, both fibroblasts and epidermal keratinocytes, by adding catalase to the culture medium, shielding the cultures from wavelengths less than 500 nm and providing a gas phase of 0-1% O2. These conditions significantly decreased the incidence of chromosomal aberrations in both cell types, and in fibroblasts prevented tumourigenicity in non-irradiated syngeneic mice, and increased latent periods for tumour development in X-irradiated mice. The epidermal keratinocytes were particularly resistant to spontaneous neoplastic transformation under all conditions tested. These observations on the protective effect of extracellular catalase suggest that H2O2, a normal metabolite, and/or the derivative .OH can directly or indirectly produce genetic damage and neoplastic transformation in mouse fibroblasts.


*Br. J. Cancer* (1985), **52**, 583–590

### Influence of added catalase on chromosome stability and neoplastic transformation of mouse cells in culture

G.M. Jones[1], K.K. Sanford[1], R. Parshad[2], R. Gantt[1], F.M. Price[1] & R.E. Tarone[3]

[1]*Laboratory of Cellular and Molecular Biology;* [3]*Biostatistics Branch, National Cancer Institute, Building 37, Room 2D15, Bethesda, Maryland 20205; and* [2]*Pathology Department, Howard University College of Medicine, Washington, DC 20059, USA.*

**Summary** The generation of hydrogen peroxide ($H_2O_2$) and the derivative free hydroxyl radical (·OH) in cultures of mouse cells grown in the presence of visible light and ambient oxygen was shown previously to be implicated in chromatid damage. Furthermore, chromosome alterations appear to be associated with the spontaneous neoplastic transformation of mouse cells in culture. An attempt was made in this study to reduce the incidence of chromosomal aberrations and delay or prevent the onset of spontaneous neoplastic transformation of freshly isolated mouse cells, both fibroblasts and epidermal keratinocytes, by adding catalase to the culture medium, shielding the cultures from wavelengths <500 nm and providing a gas phase of 0–1% $O_2$. These conditions significantly decreased the incidence of chromosomal aberrations in both cell types, and in fibroblasts prevented tumourigenicity in non-irradiated syngeneic mice, and increased latent periods for tumour development in X-irradiated mice. The epidermal keratinocytes were particularly resistant to spontaneous neoplastic transformation under all conditions tested. These observations on the protective effect of extracellular catalase suggest that $H_2O_2$, a normal metabolite, and/or the derivative ·OH can directly or indirectly produce genetic damage and neoplastic transformation in mouse fibroblasts.

The introduction of banding techniques for the analysis of chromosomes with recent developments in molecular biology has revealed chromosome aberrations in most human neoplasms and nonrandom translocations in certain mouse and human tumours that appear to activate cellular oncogenes (Land *et al.*, 1983; Potter *et al.*, 1984; Yunis, 1983). Chromosomal aberrations such as translocations, deletions and duplications may also play a role in the spontaneous neoplastic transformation of rodent cells in culture. Cells from diverse normal tissues of the mouse, rat, Syrian hamster and Chinese hamster are known to undergo spontaneous neoplastic transformation after variable periods of culture (Sanford, 1965; Sanford & Evans, 1982). The transformed cells grow as malignant neoplasms when implanted in syngeneic or compatible hosts. Such transformations, though rare, have also been reported in human cells (Mukherji *et al.*, 1984; Nakagomi & Ishida, 1980).

In 1958, Levan and Biesele showed that chromosomal irregularities precede the onset of malignancy in cultures of mouse cells and suggested a causal relation between the two phenomena. Numerous subsequent studies sought but failed to obtain conclusive evidence for such a relationship or the identification of a specific chromosomal alteration in spontaneous mouse or rat transformations in culture (Jackson *et al.*, 1970; Sanford *et al.*, 1970). However, culture conditions that accelerate or increase the incidence of neoplastic transformation in mouse cells also increase the frequency of chromosomal aberrations. These conditions include the type of serum used to supplement the culture medium, i.e., horse as compared with foetal bovine serum (Evans & Andresen, 1966; Evans & Sanford, 1978), repeated exposure of cultures to cool-white fluorescent light and a relatively high concentration of dissolved oxygen (Parshad & Sanford, 1971; Sanford *et al.*, 1979; Cooper *et al.*, 1982). Furthermore, in comparing cells of different species, including the mouse, hamster, rat and man, the incidence of spontaneous neoplastic transformation appears to correlate with chromosomal instability in culture. The frequency of transformation and the magnitude of genetic damage in cells from these different species may reflect differences in susceptibility to chromosomal DNA damage or in capacity for its repair.

The combined impact of light and oxygen on cells in culture generates photo-products including hydrogen peroxide ($H_2O_2$) (Wang & Nixon, 1978; Taylor & Camalier, 1982) which is also a metabolite (Chance *et al.*, 1979); $H_2O_2$, in turn, may give rise to the clastogenic free hydroxyl radical (·OH) through the Fenton reaction (Singh,

Correspondence: K.K. Sanford
Received 1 February 1985; and in revised form 22 May 1985.

© The Macmillan Press Ltd., 1985

1982). The chromatid breaks and interchanges seen in the first metaphase following exposure of mouse or human cells to fluorescent light (effective wavelength 405 nm in the visible range) can be almost completely prevented by adding catalase to the cultures during light exposure, an observation implicating $H_2O_2$ as a causative agent (Parshad *et al.*, 1978, 1979, 1980*b*). Intra-cellular catalase activity is known to decrease markedly soon after initiation of cells in culture (Peppers *et al.*, 1960; Parshad *et al.*, 1980*a*). Mannitol, a scavenger of •OH, also significantly decreases the chromatid damage (Parshad *et al.*, 1980*b*). In view of these observations, we have attempted, in the present study, to reduce the incidence of chromosomal aberrations and delay or prevent the onset of spontaneous neoplastic transformation of freshly isolated mouse cells, both fibroblasts and epidermal keratinocytes, by adding catalase to the culture medium, shielding the cultures from wavelengths $<500$ nm and providing a gas phase of 0–1% $O_2$.

### Materials and methods

### *Cells and culture procedures*

Embryo cell lines were initiated from 4 pools of minced 11–12 day $C3H_f$/HeN or C3H/HeN-MTV$^-$ embryos. Cultures were carried in Pyrex T-15 flasks in 3 ml Dulbecco's modified Eagle's medium (DMEM, MA Bioproducts, Walkersville, MD) supplemented with 10% foetal bovine serum (Flow Laboratories, Inc., McLean, VA, USA). Pyrex rather than plastic flasks were used for more precise control of dissolved oxygen ($PO_2$) (Chapman & Sturrock, 1969). Medium was renewed three times weekly when cultures were gassed with a humidified mixture of 10% $CO_2$ in air (18% $O_2$) except as indicated. No antibiotics were used and cultures were negative for mycoplasma by direct and indirect tests (Flow Laboratories). Cultures were passaged (1:2 or 1:4), usually at weekly intervals, by an initial rinse with versene (1:5000, M.A. Bioproducts) in $Ca^{++}Mg^{++}$-free saline, followed by cell dispersion with 0.1% trypsin (3 × crystallized, Worthington, Freehold, NJ). Except as indicated, cultures and medium in this laboratory were not exposed to light of wavelength $<500$ nm since all manipulations were carried out under gold or red light.

A line of epidermal keratinocytes, NCTC 10313, was initiated from 1 day-old $C3H_f$/HeN mice essentially by the method of Hennings *et al.* (1980) and was cultured as above except in T-25 plastic flasks containing 5 ml $Ca^{++}$-free Eagle's MEM with nonessential amino acids (M.A. Bioproducts) supplemented with 8% Chelex-treated foetal bovine serum (FBS) (Flow Laboratories, Inc.) and 0.1 mg ml$^{-1}$ garamycin (Schering Corp., Kenilworth, NJ) to yield a [$Ca^{++}$] of 0.02 mM.

### *Assay for neoplastic transformation*

A suspension of $10^6$ to $10^7$ cells per 0.25–1 ml culture medium was injected into thigh muscles of syngeneic mice which had been irradiated (whole-body dose, 4.25 Gy) except as indicated. Mice were palpated at weekly intervals for tumours and observed for 5 to 8 months (embryo cells) or 10–12 months (keratinocytes). Tumour sections were fixed in Zenker-Formol, stained with haematoxylin and eosin and diagnosed as sarcomas. Mice that failed to develop tumours during the experimental period were sacrificed and tissue from the injection site fixed and stained as above for microscopic diagnosis. Tumour latent periods measured the interval from time of injection to appearance of a palpable tumour. Statistical evaluation of tumour latent periods was performed using the Wilcoxon Rank Sum test (Snedecor & Cochran, 1980).

### *Chromosome preparation and analysis*

Cells ($5 \times 10^4$) in 2 ml of medium were inoculated into Leighton tubes, each containing a 9 × 50 mm No. 1 coverslip. After 48 h incubation at 37°C, colcemid (0.1 $\mu$g ml$^{-1}$) was added for 1 h of additional incubation. Cells were processed *in situ* on coverslips by techniques described (Gantt *et al.*, 1978). Analyses were made on randomized, coded preparations; 4 cultures were used for each variable, and 100–200 metaphase cells were studied per variable. Abnormalities scored as chromatid breaks show distinct dislocation and misalignment of the chromatid fragment, whereas chromatid gaps are achromatic lesions, longer than the chromatid width, with no dislocation of the segment distal to the lesion. Minutes are chromosomes less than one half the length of the shortest chromosome of the mouse karyotype.

### *Experimental procedures*

Cells were grown with and without exogenous catalase, intermittent light exposure and 0% $O_2$ to assess their influence on chromosome stability and neoplastic transformation. As indicated (Figure 1), two lines of fibroblasts were initiated from each embryo cell pool, one with a supplement of catalase (hydrogen peroxide: hydrogen peroxide oxido-reductase EC 1.11.1.6). A stock solution of catalase (1 mg ml$^{-1}$, Sigma, 13,000 U mg$^{-1}$) was stored at −20°C in small aliquots, thawed for single use only, and 10 $\mu$g ml$^{-1}$ added to the cultures at each fluid renewal. Some cultures of cell pool IV (Figure 1) were grown with a gas phase of 0% $O_2$: 10% $CO_2$: 90% $N_2$ which yields an initial dissolved $O_2$

**Figure 1** Relationships of cell lines derived from the four mouse embryo cell pools. ↗ = catalase ($10 \mu g\, ml^{-1}$); hv = $5\, w\, m^{-2}$ light; * = 0% $O_2$.

concentration ($PO_2$) of 20–40 mm Hg. All other embryo cell lines were initiated and carried continuously in growth medium equilibrated with 10% $CO_2$ in air (18% $O_2$) which yields an initial $PO_2$ of ~120 mm Hg (Taylor *et al.*, 1979; Taylor & Camalier, 1982). Some cells were transferred from 0% to 18% $O_2$ (Figure 1) at the 14th passage (182–187 days in culture), and catalase treatment was discontinued.

As indicated (Figure 1) certain cultures of cell pool III were exposed once a week for 24 h at 37°C directly after medium renewal to cool-white fluorescent light (Westinghouse F15T8-CW); light intensity was $5\, W\, m^{-2}$ at the growth surface measured by an IL 700 Research Radiometer (International Light, Inc., Newburyport, MA, USA). Certain cultures of cell pool IV were exposed to light as above twice a week for 10 h directly after medium renewal.

A subline of the epidermal keratinocytes was carried with exogenous catalase ($10 \mu g\, ml^{-1}$) during all but the first passage in culture. A second subline was carried with exogenous catalase from the time of recovery from liquid nitrogen. Some cultures were exposed weekly to 5 h fluorescent light as described above.

### *Catalase activity*

The effect of fluorescent light ($5\, W\, m^{-2}$) on catalase activity ($10 \mu g\, ml^{-1}$) at 37°C in culture medium (15 ml DMEM with 10% foetal bovine serum in a Pyrex T-60 flask) or in PBS (0.01 M sodium phosphate, 0.9% NaCl, pH 7.2) with riboflavin ($0.4 \mu g\, ml^{-1}$) was determined. At various times during exposure, a 0.5 ml sample was removed, added to 1.5 ml PBS in a 3 ml cuvette (10 mm light path) and an absorbance (254 nm) baseline established with a Gilford model 240 recording spectrophotometer. Then $10 \mu l$ of 30% hydrogen peroxide was added to the cuvette with rapid mixing which increased the absorbance ~0.990 units. Enzyme activity was determined from slope of the absorbance decline between 0.700 and 0.600 units.

### Results

### *Chromosomal aberrations*

The addition of catalase to the cultures of mouse embryo fibroblasts from the time of initiation significantly reduced the incidence of chromosomal aberrations (Table I). The pattern for each pair of cell lines with and without catalase was similar. Differences in minutes and metacentrics were more significant at later passages, whereas differences in chromatid breaks and gaps were more significant at earlier passages when each of these types of aberrations is more likely to occur. The incidence of

**Table I** Influence of catalase and oxygen on chromosomal aberrations in mouse fibriblasts

| *Embryo cell pool* | *NTC cell line* | *Passage* | *Catalase (10 μg ml⁻¹)* | *18% O₂* | *Average number/100 cells*[a] *Minutes* | *Metacentrics* | *Interchanges* | *Chromatid breaks/gaps* |
|---|---|---|---|---|---|---|---|---|
| 1 | 10295 | 5 | − | + | 3 | 5 | 0 | 10 |
| | | 14 | − | + | 3 | 1 | 0 | 4 |
| | | 18 | − | + | 4 | 5 | 0 | 0 |
| | | 25 | − | + | 8 | 14 | 0 | 1 |
| | 10296 | 6 | + | + | 3 | 1 | 0 | 4 |
| | | 14 | + | + | 0 | 0 | 0 | 3 |
| | | 18 | + | + | 0 | 2 | 0 | 0 |
| | | 24 | + | + | 2 | 10 | 0 | 0 |
| | | | | | $P=0.018$[b] | $P=0.12$ | $P>0.50$ | $P=0.16$ |
| 2 | 10297 | 6 | − | + | 5[c] | 1 | 1 | 13 |
| | | 20 | − | + | 16 | 11 | 3 | 5 |
| | | 23 | − | + | 21 | 18 | 0 | 2 |
| | 10298 | 6 | + | + | 0 | 1 | 0 | 1 |
| | | 19 | + | + | 3 | 1 | 0 | 0 |
| | | 22 | + | + | 12 | 10 | 0 | 0 |
| | | | | | $P=0.001$ | $P=0.025$ | $P>0.50$ | $P=2\times10^{-4}$ |
| 3 | 10299 | 6 | − | + | 8 | 5 | 1 | 16 |
| | | 12 | − | + | 12 | 6 | 1 | 6 |
| | | 19 | − | + | 5 | 2 | 0 | 17 |
| | | 24 | − | + | 31 | 20 | 0 | 4 |
| | | 36 | − | + | 31 | 38[d] | 0 | 3 |
| | 10300 | 6 | + | + | 2 | 3 | 0 | 0 |
| | | 13 | + | + | 1 | 0 | 0 | 1 |
| | | 19 | + | + | 0 | 1 | 0 | 2 |
| | | 24 | + | + | 6 | 6 | 0 | 0 |
| | | 36 | + | + | 8 | 18[e] | 0 | 0 |
| | | | | | $P<10^{-6}$ | $P=3\times10^{-5}$ | $P>0.50$ | $P<10^{-6}$ |
| 4 | 10705 | 5 | − | + | 14 | 26 | 1 | 3 |
| | 10706 | 5 | + | − | 1 | 4 | 0 | 0 |
| | | | | | $P=10^{-6}$ | $P<10^{-6}$ | $P>0.50$ | $P=0.060$ |

[a]In each analysis 100 to 200 metaphase cells were examined with 2 exceptions: NCTC line 10299 passage 12 (78 cells); line 10298 passage 19 (74 cells); [b]Summary *P* values were obtained using the Mantel-Haenszel statistic (Snedecor and Cochran) combining the information from all passages. For each passage, the analysis was based on the 2×2 table comparing the proportion of cells with one or more aberrations in cultures with and without catalase; [c]One additional cell with 6 minutes; [d]Two cells with one long acrocentric each; [e]One cell with a long acrocentric.

chromatid interchanges was minimal. In the experiment on pool #4 (Table I), catalase and a supernatant gas mixture of 0% $O_2$ produced only minimal chromosomal aberrations compared with the high incidence in cells initiated and carried continuously without catalase and with a gas mixture containing 18% $O_2$ (90% air: 10% $CO_2$). In a number of previous studies, high $PO_2$ compared with low $PO_2$ in the absence of added catalase was found to increase the frequency of chromosomal aberrations (Parshad & Sanford, 1971; Parshad *et al.*, 1977; Sanford *et al.*, 1979). Catalase did not prevent the development of heteroploidy.

In two experiments on epidermal keratinocytes, addition of catalase was associated with a decreased incidence of both metacentric ($P<10^{-6}$) and minute ($P=0.025$) chromosomes when results of the experiments were combined and analyzed by the Mantel-Haenszel test (Snedecor & Cochran, 1980) (Figure 2). Again, catalase did not prevent development of heteroploidy; cell populations were heteroploid by the first analysis after 13 passages (28 weeks) in culture.

The efficacy of catalase in reducing chromatid damage implicates generation of $H_2O_2$ as a direct or indirect cause of chromosomal aberrations in both mouse fibroblasts and epidermal cells.

**Figure 2** Chromosomal aberrations in keratinocyte cell lines carried with and without exogenous catalase. In Experiment A, a subline of NCTC 10313 was carried with exogenous catalase during all but the first passage in culture; 83 and 100 metaphase figures were examined of control and treated cultures, respectively. In Experiment B, a subline of NCTC 10313 was carried with exogenous catalase from the time of recovery from liquid nitrogen; 45 and 39 metaphase cells were examined of control and treated cultures, respectively. □ = chromatid breaks; ■ = minutes; ▨ = metacentrics.

### *In vivo assays*

Cells were implanted *in vivo* to determine whether addition of catalase, intermittent exposure to light, or lowered $O_2$ tension influenced neoplastic transformation. In the first experiment (Table II), mouse embryo fibroblasts cultured with or without catalase grew as sarcomas in all X-irradiated syngeneic mice when first injected at the 42nd passage. However, the tumour latent periods for cells grown with catalase and shielded from light were significantly longer ($P = 2 \times 10^{-5}$). The tumour latent periods were also significantly longer than those of cells grown with catalase but repeatedly exposed to light ($P = 0.027$). Repeated exposure of cells to light in the absence of catalase did not further increase tumourigenicity as measured by a shortened tumour latent period.

In the second experiment (Table II) cells grown with catalase and 0% oxygen failed to produce tumours in nonirradiated syngeneic mice but did grow as sarcomas after a prolonged latent period in some but not all X-irradiated mice. In contrast, cells cultured without catalase and with 18% oxygen grew as sarcomas in both non-irradiated and/or X-irradiated mice. Tumour latent periods for sublines of NCTC line 10706 were significantly shorter in both non-irradiated and X-irradiated mice than observed for cells grown with catalase and 0% oxygen ($P < 10^{-5}$). Repeated exposure of cells to light in the absence of catalase did not further increase tumourigenicity as measured by a shortened tumour latent period. However, an influence of light exposure on tumourigenicity has been observed previously (Sanford *et al.*, 1979). Addition of catalase, lowered $O_2$ tension, and/or light shielding thus appear to delay or prevent the onset of tumourigenicity in mouse fibroblasts.

In contrast to mouse embryo fibroblasts, mouse epidermal keratinocytes under the culture conditions of this study appeared refractory to neoplastic transformation. Cells were assayed in X-irradiated syngeneic mice after 47 and 56 weeks in culture. In each assay, 5 mice were implanted with (i) untreated cells, (ii) cells treated with catalase from the 13th week in culture, (iii) cells grown with catalase and exposed weekly to 5 h fluorescent light from the 43rd week in culture, and (iv) cells grown without catalase and light exposed as in (iii). No tumours developed in the total of 40 mice implanted.

### *Catalase activity*

The failure of catalase to provide complete protection for light-exposed cultures suggested that catalase activity might be unstable in the presence of light. Catalase activity in culture medium exposed to fluorescent light at 37°C was found to decline, whereas in light-shielded medium activity remained relatively high (Figure 3). A similar rate of decline in catalase activity was observed when light-exposed in a saline solution containing riboflavin at the concentration in DMEM; riboflavin has been shown to be a photosensitizing agent in culture medium (Wang & Nixon, 1978) and is probably responsible for the inactivation of added catalase. A 5-fold increase in riboflavin in either saline or medium did not further increase the rate of inactivation, which appeared, therefore, to be maximal by $0.4\,\mu g\,ml^{-1}$.

**Table II** Results of assays of fibroblasts in syngeneic mice

| *NCTC cell line and subline* | *Days in culture* | *Passage* | *Treatment* | | | *X-irradiation of host (4.25 Gy)* | *Mice with tumours/number injected* | *Average period days (range)* |
|---|---|---|---|---|---|---|---|---|
| | | | *Catalase* | *0% $O_2$* | *Light 5 $Wm^{-2}$* | | | |
| Expt. 1 | | | | | | | | |
| 10299 | 315 | 42 | – | – | – | + | 10/10 | 45 (29–49) |
| 10300 | 315 | 42 | + | – | – | + | 9/9 | 80 (57–98) |
| 10299-A[a] | 315 | 43 | – | – | + | + | 10/10 | 49 |
| 10300-A[a] | 315 | 43 | + | – | + | + | 8/8 | 54 (41–95) |
| Expt. 2 | | | | | | | | |
| 10705 | 217 | 22 | – | – | – | + | 3/3 | 67 |
| 10706-A | 281 | 24 | – | – | – | – | 5/5 | 43 (35–45) |
| | 297 | 27 | – | – | – | + | 5/5 | 38 |
| | 280 | 26 | – | – | – | – | 5/5 | 52 (50–55) |
| B | 296 | 29 | – | – | – | + | 5/5 | 42 (39–43) |
| C[b] | 297 | 28 | – | – | + | + | 5/5 | 33 |
| D[b] | 296 | 27 | – | – | + | + | 5/5 | 43 (39–50) |
| E | 217 | 16 | + | + | – | + | 0/3 | |
| F | 281 | 25 | + | + | – | – | 0/5 | |
| | 297 | 27 | + | + | – | + | 5/5 | 97 (77–109) |
| G | 280 | 24 | + | + | – | – | 0/5 | |
| | 296 | 27 | + | + | – | + | 4/5 | 78 |

[a]Light exposure at 37°C 24 h weekly for 69 days before injection; [b]Light exposure at 37°C for 10 h 2 × weekly for 88 or 105 days before injection.

**Figure 3** Effect of fluorescent light on catalase activity in DMEM with 10% foetal bovine serum exposed (○), and unexposed (●) to light; catalase in phosphate-buffered saline with riboflavin exposed (□) and unexposed to (■) light. Bar = ± standard deviation.

### Discussion

The main finding of this study was that addition of catalase to cultures of freshly isolated mouse embryonic fibroblasts shielded from light and grown with a gas phase of 0% oxygen, significantly reduced the incidence of chromosomal aberrations, prevented tumourigenicity of cells implanted in non-irradiated syngeneic mice and increased latent periods for tumour development in X-irradiated mice. Catalase also significantly reduced the incidence of chromosome aberrations in mouse epidermal keratinocytes. A prolonged latent period conceivably results from a small proportion of neoplastic cells in the implanted cell population, from cells of low malignancy or from immunologic incompatibility between cultured cells and recipient host tissues. Where comparisons can be made, tumour latent periods were somewhat shorter in X-irradiated than in non-irradiated mice implanted with either catalase-treated or untreated cells, an observation suggesting some immunologic incompatibility between culture cells and host. Nevertheless, the results of implants into non-irradiated and X-irradiated syngeneic mice support the conclusion that extracellular catalase delayed or suppressed the onset of neoplastic transformation.

It thus appears that the generation of $H_2O_2$ is a direct or indirect agent in chromosomal DNA damage and spontaneous neoplastic transformation. $H_2O_2$, in turn, may give rise to the clastogenic ·OH as suggested by the effectiveness of mannitol, an ·OH scavenger, in reducing light-induced chromosomal damage (Parshad *et al.*, 1980*b*, 1982*b*). The $H_2O_2$ may arise endogenously from metabolic processes or be generated intracellularly or in culture medium on exposure of cells or medium to visible light and ambient $O_2$ (Parshad *et al.*, 1980*b*; Wang & Nixon, 1978). In view of the low catalase activity of mouse cells in culture (Parshad *et al.*, 1980*a*; Peppers *et al.*, 1960), the generation of $H_2O_2$ may be a source of cell injury and chromosomal DNA damage. Although exogenous catalase can readily enter the cell through pinocytosis, it probably acts to destroy $H_2O_2$ passing freely between cells and culture medium.

In contrast to fibroblasts, the epidermal keratinocytes, though showing chromosomal aberrations, were particularly resistant to spontaneous neoplastic transformation, possibly because of their less rapid cell cycling during early *in vitro* growth and their adjustment of the dissolved $O_2$ concentration of the culture medium (Taylor & Camalier, 1982; Taylor, unpublished observations). Since lesions introduced into DNA during S phase, if unrepaired, may lead to DNA replication errors, rapid cell cycling may increase the risk of fixation of genetic alterations and ultimate neoplastic transformation. Furthermore, the more rapid consumption of $O_2$ by mouse epidermal keratinocytes than fibroblasts maintains an environmental $PO_2$ comparable to intercellular fluids *in vivo* and thus tends to mitigate oxidative cell injury and genomic damage.

It should be noted that catalase did not prevent the development of heteroploidy which can result from deficient spindle function, incomplete cytokinesis or cell fusion (McQuilkin & Earle, 1962). As noted earlier, mouse cells, in contrast to human cells in culture, show chromosomal instability and quite regularly undergo spontaneous neoplastic transformation. They are also reportedly less efficient in nucleotide excision repair of DNA damage (Yagi, 1982). Furthermore, they tend to undergo a tetraploid shift during early culture, a process that may be important for cell survival (Parshad *et al.*, 1968). Because of the tetraploid complement of chromosomes and the development of heteroploidy, mouse cells may survive loss, deletions and translocations of chromosomes that in human diploid cells would be lethal. These unique properties of mouse cells may account for their high rate of spontaneous neoplastic transformation in culture.

### References

CHANCE, B., SIES, H. & BOVERIS, A. (1979). Hydroperoxide metabolism in mammalian organs. *Physiol. Rev.*, **59**, 527.

CHAPMAN, J.C., STURROCK, J. (1969). The oxygen tension around mammalian cells growing on plastic Petri dishes and its effect on cell survival curves. *Br. J. Radiol.*, **42**, 339.

COOPER, P.D., MARSHALL, S.A. & MASINELLA, G.R. (1982). Rapid induction of foci escaping density-dependent inhibition in baby mouse skin cultures. *J. Cell. Physiol.*, **113**, 329.

EVANS, V.J., & ANDRESEN, W.F. (1966). Effect of serum on spontaneous neoplastic transformation *in vitro*. *J. Natl Cancer Inst.*, **37**, 247.

EVANS, V.J. & SANFORD, K.K. (1978). Development of defined media for studies on malignant transformation in culture. In *Nutritional Requirements of Cultured Cells*, p. 149 (Ed. Katsuta.) University Park Press: Baltimore.

GANTT, R., PARSHAD, R., EWIG, R.A.G. & 4 others. (1978). Fluorescent light-induced DNA crosslinkage and chromatid breaks in mouse cells in culture. *Proc. Natl Acad. Sci.* (*USA*) **75**, 3809.

HENNINGS, H., MICHAEL, D., CHENG, C., STEINERT, P., HOLBROOK, K. & YUSPA, S.H. (1980). Calcium regulation of growth and differentiation of mouse epidermal cells in culture. *Cell*, **19**, 245.

JACKSON, J.L., SANFORD, K.K. & DUNN, T.B. (1970). Neoplastic conversion and chromosomal characteristics of rat embyro cells *in vitro*. *J. Natl Cancer Inst.* **45**, 11.

LAND, H., PARADA, L.F. & WEINBERG, R.A. (1983). Cellular oncogenes and multistep carcinogenesis. *Science*, **222**, 771.

LEVAN, A. & BIESELE, J.J. (1958). Role of chromosomes in cancerogenesis as studied in serial tissue culture of mammalian cells. *Ann. N.Y. Acad. Sci.*, **71**, 1022.

McQUILKIN, W.T. & EARLE, W.R. (1962). Cinemicrographic analysis of cell populations in vitro. *J. Natl Cancer Inst.*, **28**, 763.

MUKHERJI, B., MacALISTER, T.J., GUHA, A., GILLIES, C.G., JEFFERS, D.C. & SLOCUM, S.K. (1984). Spontaneous *in vitro* transformation of human fibroblasts. *J. Natl Cancer Inst.*, **73**, 583.

NAKAGOMI, O. & ISHIDA, N. (1980). Establishment of a cell line from a human fetal liver and its xenotransplantation to nude mice. *Gann*, **71**, 213.

PARSHAD, R., GANTT, R., SANFORD, K.K., JONES, G.M. & TARONE, R.E. (1982*a*). Repair of chromosome damage induced by X-irradiation during $G_2$ phase in a line of normal human fibroblasts and its malignant derivative. *J. Natl Cancer Inst.*, **69**, 404.

PARSHAD, R. & SANFORD, K.K. (1968). Effect of horse serum, fetal calf serum, calf serum, bovine serum, and fetuin on neoplastic conversion and chromosomes of mouse embryo cells *in vitro*. *J. Natl Cancer Inst.*, **41**, 767.

PARSHAD, R. & SANFORD, K.K. (1971). Oxygen supply and stability of chromosomes in mouse embryo cells *in vitro*. *J. Natl Cancer Inst.*, **47**, 1033.

PARSHAD, R., SANFORD, K.K., JONES, G.M., PRICE, F.M. & TAYLOR, W.G. (1977). Oxygen and light effects on chromosomal aberrations in mouse cells *in vitro*. *Exp. Cell Res.*, **104**, 199.

PARSHAD, R., SANFORD, K.K., JONES, G.M. & TARONE, R.E. (1978). Fluorescent light-induced chromosome damage and its prevention in mouse cells in culture. *Proc. Natl Acad. Sci.* (*USA*), **75**, 1830.

PARSHAD, R., SANFORD, K.K., JONES, G.M., TARONE, R.E., HOFFMAN, H.A. & GRIER, A.H. (1980*a*). Susceptibility to fluorescent light-induced chromatid breaks associated with DNA repair deficiency and malignant transformation in culture. *Cancer Res.*, **40**, 4415.

PARSHAD, R., SANFORD, K.K., JONES, G.M. & TARONE, R.E. (1982*b*). Neoplastic transformation of human cells in culture associated with deficient repair of light-induced chromosomal DNA damage. *Int. J. Cancer*, **30**, 153.

PARSHAD, R., SANFORD, K.K., TAYLOR, W.G., TARONE, R.E., JONES, G.M. & BAECK, A.E. (1979). Effect of intensity and wavelength of fluorescent light on chromosome damage in cultured mouse cells. *Photochem. Photobiol.* **29**, 971.

PARSHAD, R., TAYLOR, W.G., SANFORD, K.K., CAMALIER, R.F., GANTT, R. & TARONE, R.E. (1980*b*). Fluorescent light-induced chromosome damage in human IMR-90 fibroblasts. *Mutat. Res.*, **73**, 115.

PEPPERS, E.V., WESTFALL, B.B., KERR, H.A. & EARLE, W.R. (1960). Note on the catalase activity of several mammalian cell strains after long cultivation *in vitro*. *J. Natl Cancer Inst.*, **25**, 1065.

POTTER, M., WIENER, F. & MUSHINSKI, F. (1984). Recent developments in plasmacytomagenesis in mice. *Adv. Viral Oncol.*, **4**, 139.

SANFORD, K.K. (1965). Malignant transformation of cells *in vitro*. *Int. Rev. Cytol.*, **18**, 249.

SANFORD, K.K., BARKER, B.E., PARSHAD, R. & 5 others. (1970). Neoplastic conversion *in vitro* of mouse cells: Cytologic, chromosomal, enzymatic, glycolytic, and growth properties. *J. Natl Cancer Inst.*, **45**, 1071.

SANFORD, K.K. & EVANS, V.J. (1982). A quest for the mechanism of 'spontaneous' malignant transformation in culture with associated advances in culture technology. *J. Natl Cancer Inst.*, **68**, 895.

SANFORD, K.K., PARSHAD, R., JONES, G., HANDLEMAN, S., GARRISON, C. & PRICE, F. (1979). Role of photosensitization and oxygen in chromosome stability and 'spontaneous' malignant transformation in culture. *J. Natl Cancer Inst.*, **63**, 1245.

SINGH, A. (1982). Chemical and biochemical aspects of superoxide radicals and related species of activated oxygen. *Can. J. Physiol. Pharmacol.*, **60**, 1330.

SNEDECOR, G.W. & COCHRAN, W.G. (1980). *Statistical Methods*, p. 208, University Press: Ames, IO.

TAYLOR, W.G., CAMALIER, R.F. & TAYLOR, M.J. (1979). A spectrophotometric assay for hydrogen peroxide in tissue culture medium. *Tissue Culture Association Manual*, **5**, 1081.

TAYLOR, W.G. & CAMALIER, R.F. (1982). Modulation of epithelial cell proliferation in culture by dissolved oxygen. *J. Cell. Physiol.*, **111**, 21.

WANG, R.J. & NIXON, B.T. (1978). Identification of hydrogen peroxide as a photoproduct toxic to human cells in tissue-culture medium irradiated with 'daylight' fluorescent light. *In Vitro*, **14**, 715.

YAGI, T. (1982). DNA repair ability of cultured cells derived from mouse embryo in comparison with human cells. *Mutat. Res.*, **96**, 89.

YUNIS, J.J. (1983). The chromosomal basis of human neoplasia. *Science*, **221**, 227.